# Cell aggregation prevents anoikis and induces CD44 cleavage by maintaining lipid raft integrity to promote triple negative breast cancer metastasis

**DOI:** 10.21203/rs.3.rs-2535728/v1

**Published:** 2023-02-14

**Authors:** dong li, younhee park, hami hemati, xia liu

**Affiliations:** university of kentucky; university of Kentucky; university of Kentucky; University of Kentucky

## Abstract

Triple-negative breast cancer (TNBC) is the most aggressive breast cancer subtype, and metastasis is the major cause of cancer morbidity and mortality. Therefore, it is urgent to discover novel therapeutic targets and develop effective treatments for this lethal disease. Circulating tumor cells (CTCs) are considered “seeds of metastasis”. Compared to single CTCs, our previous studies have demonstrated that CD44 homophilic interaction mediates CTC aggregation to enhance the stemness, survival and metastatic ability of aggregated cells. Importantly, the presence of CD44+ CTC clusters correlates with a poor prognosis in breast cancer patients. Here, we further investigated the underlying mechanism of how CD44-mediated cell aggregation promotes TNBC metastasis. We found that cell detachment, which mimics the condition when tumor cells detach from the extracellular matrix (ECM) to metastasize, induces lipid raft disruption in single cells, but lipid rafts integrity is maintained in aggregated cells. We further found that lipid rafts integrity in aggregated cells is required for Rac1 activation to prevent anoikis. In addition, CD44 and γ-secretase coexisted at lipid rafts in aggregated cells, which promotes CD44 cleavage and generates CD44 intracellular domain (CD44 ICD) to enhance stemness. Consequently, lipid rafts disruption inhibited Rac1 activation, CD44 ICD generation and metastasis. These data reveal a new mechanism of cell aggregation-mediated TNBC metastasis via maintaining lipid raft integrity after cell detachment. The finding provides a potential therapeutic strategy to prevent CTC cluster-initiated metastasis by disrupting lipid raft integrity and its-mediated downstream pathways.

## 1. Introduction

Metastasis is responsible for the majority of cancer-related patient deaths. Triple-negative breast cancer (TNBC), which accounts for 15–20% of all breast cancers, is the most aggressive breast cancer subtype, and the only subtype without established targeted therapy^1^. Therefore, it is urgent to discover novel therapeutic targets, and develop effective treatments for this lethal disease. Our recent studies and others have demonstrated that circulating tumor cell (CTC) clusters (defined as groups of two or more aggregated CTCs) have higher metastatic potential than single CTCs, and correlate with worse prognosis^2–4^. Therefore, targeting CTC cluster provides a new opportunity to prevent metastatic spread.

To metastasize, cancer cells have to detach from the primary tumor, and travel to different sites. Normally, cells undergo apoptosis after losing contact with their extracellular matrix(ECM), which has been termed “anoikis” and is a barrier to metastasis^5^. However, some cancer cells can develop mechanisms to resist anoikis and thereby surviving after detachment from their primary site, giving rise to metastasis^6–10^. Thus, a better understanding of how cancer cells survive after detachment will provide the basis for developing therapeutics to eliminate metastasizing cancer cells. Lipid rafts are highly ordered membrane domains that are enriched in cholesterol and glycosphingolipids, and serve as major platforms for signal transduction^11–13^. It has been reported that cell detachment from the ECM triggers the internalization of lipid rafts (or lipid rafts disruption) and anoikis^5,14^. Recently, we found that CD44-mediated cell aggregation can prevent cells from anoikis after cell detachment^2^. We also found that CD44-mediated cell aggregation activates Pak2 (the p21 activated kinase 2)^2,4^, a downstream target of Rac1^15^. Since Rac1 activation can prevent epithelial cells from anoikis^16,17^, and previous studies have shown that lipid rafts integrity is required for maintaining Rac1 membrane targeting and effector activation in ECM-detached cells^18^, raising a possibility that CD44-mediated cell aggregation may maintain lipid rafts integrity to activate Rac1-Pak2 pathway to prevent anoikis.

CD44 is a well-known breast cancer stem cell marker^19,20^. Proteolytic cleavages of CD44 is a regulatory mechanism for the CD44-mediated signaling pathways^21,22^. CD44 is cleaved by matrix metallopeptidases to generate the membrane-bound C-terminal fragment of CD44 (CD44 EXT), followed by γ-secretase to generate CD44 intracellular domain (CD44 ICD)^21–26^. CD44 ICD then acts as a signal transduction molecule, and is translocated to the nucleus and activates genes transcription. Among these genes, CD44 ICD can activate stemness factors such as Oct4 to enhance tumor cell stemness and promote tumorigenesis^26–28^. Both CD44 and γ-secretase are present in lipid rafts^29–31^, and we have found that aggregated CD44^+^ TNBC cells have enhanced stemness by upregulating Oct4^2^, suggesting lipid rafts may be involved in enhanced stemness of CD44-mediated cell aggregation.

In this study, we reveal a new mechanism of cell aggregation-mediated TNBC metastasis, that is cell aggregation maintains lipid raft integrity after cell detachment to prevent anoikis via activating Rac1 and enhance stemness by promoting CD44 cleavage. The finding provides a potential therapeutic strategy to prevent CTC cluster-initiated metastasis by disrupting lipid raft integrity and its-mediated downstream pathways.

## 2. Results

### 2.1. Cell aggregation maintains lipid rafts integrity after cell detachment.

**Figure 1:**
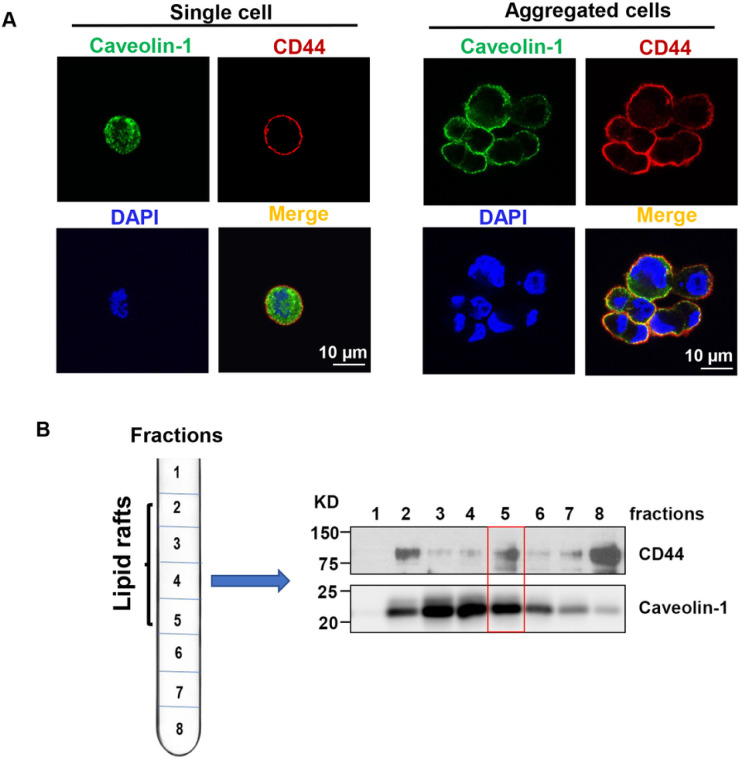
CD44-mediated cell aggregation maintains lipid rafts integrity after cell detachment. **A.** Immunofluorescence staining shows that CD44-mediated cell aggregation maintains lipid rafts integrity in aggregated cells (left), but not the single cells (right) after cell detachment. The MDA-MB-231 cells were cultured in poly-HEMA-coated dishes to aggregate for 24 hours, and then cells were dry onto cover slides for IF staining. Red: CD44; Green: caveolin-1 (lipid raft marker); Blue: DAPI. **B.**CD44 colocalizes with caveolin-1 at lipid rafts. The MDA-MB-231 cells were cultured in poly-HEMA-coated dishes to aggregate for 24 hours, and then lipid rafts were isolated using Caveolae/Rafts Isolation Kit (the lipid rafts are enriched in 2–5 fractions showing high caveolin-1 expression). The colocalization of CD44 with caveolin-1 at fraction 5 was indicated in red box. The data represent one of three independent experiments.

Cell detachment changes the structure of the cell membrane, and affects the activities of many downstream signaling pathways. Lipid rafts are highly ordered membrane domains that serve as major platforms for signal transduction^11–13^. When MDA-MB-231 cells were cultured in the poly-HEMA-coated dishes that prevent cells adhesion and force them to grow in suspension (which mimics the condition of tumor cells detachment from the ECM), we found that caveolin-1 (the marker for lipid rafts) is delocalized from cell membrane into the cytoplasm, suggesting that lipid rafts are disrupted in the single cells (Fig. 1A).Surprisingly, the lipid raft integrity (caveolin-1 mainly localizes on the cell membrane) was maintained in aggregated CD44^+^ cells (Fig. 1A). In addition, we found that CD44 colocalizes with cavelin-1 on cell membranes in aggregated cells (Fig. 1A). Since cavelin-1 is a marker for lipid rafts, we further determined whether CD44 colocalizes with cavelin-1 in lipid rafts. We isolated lipid rafts from aggregated MDA-MB-231 cells after suspension culture for 24 hours, and found that CD44 was highly expressed and coexisted with caveolin-1 in fraction 5 (according to the instruction of the isolation kit, the lipid rafts are enriched in 2–5 fractions, Fig. 1B), suggesting CD44 localizes at lipid rafts in the aggregated cells. Collectively, these data suggest that cell detachment induces lipid rafts disruption in single cells, but lipid rafts integrity is maintained in aggregated cells.

### 2.2. Disruption of lipid rafts induces aggregated cells to undergo anoikis.

**Figure 2:**
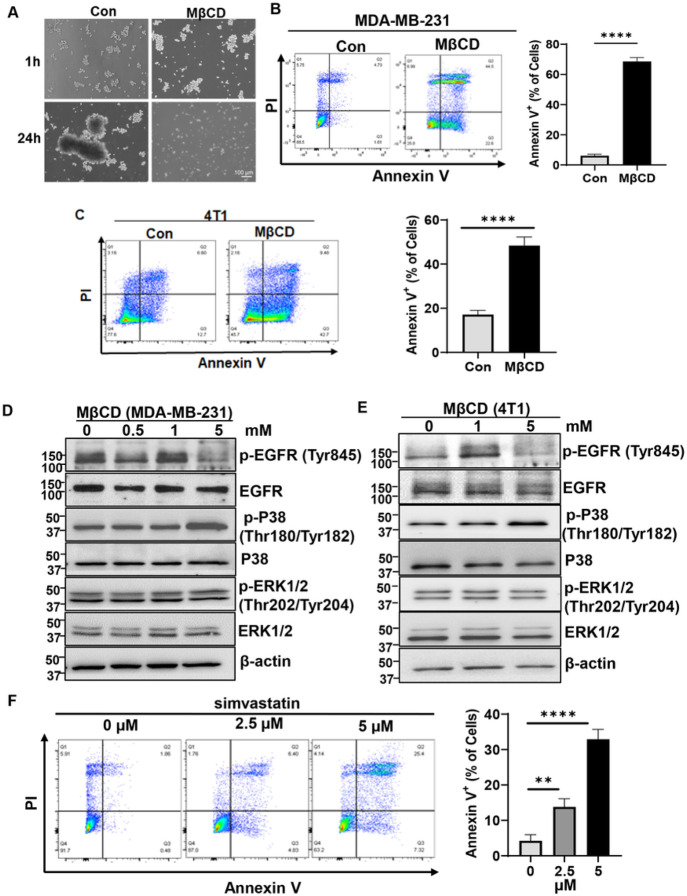
Disruption of lipid rafts promotes aggregated cells to undergo anoikis. **A.** Representative images of cell aggregation of MDA-MB-231 cells cultured in poly-HEMA-coated dishes in the absence or presence of MβCD (5mM, pretreatment for 1 hour) for the indicated time points. **B&C.** Disruption of lipid rafts by MβCD promotes anoikis. The MDA-MB-231 cells (B) and 4T1 cells (C) were cultured in poly-HEMA-coated dishes in the presence or absence of MβCD (5mM) for 24 hours, and the cells were collected for anoikis analysis by Annexin V/PI staining (left). The percentage of Annexin V^+^ cells were calculated (right). Graph data were presented as mean ± SEM (n=3). T-test, ****p<0.001. **D&E.** Western blotting analysis of p-EGFR, p-p38 and p-ERK expression in MDA-MB-231 cells (D) and 4T1 cells (E) cultured in poly-HEMA-coated dishes in the presence or absence of MβCD for 24 hours. β-actin serves as a loading control. The data represent one of two independent experiments. **F.** The effect of simvastatin on anoikis. The MDA-MB-231 cells were cultured in poly-HEMA-coated dishes in the presence or absence of simvastatin for 48 hours, and the cells were collected for anoikis analysis by Annexin V/PI staining. The percentage of Annexin V^+^ cells were calculated (right). Graph data were presented as mean ± SEM (n=3). T-test, **p<0.05, ****p<0.001.

Normally, cells undergo anoikis after they lose contact with ECM. But we found that CD44-mediated cell aggregation can prevent anoikis after cell detachment^2^. Since we have found that cell aggregation can maintain lipid rafts integrity, and previous studies demonstrated that lipid raft integrity is required for the survival of TNBC cells^32^, we examined whether disruption of lipid rafts could induce anoikis in aggregated cells. We pretreated MDA-MB-231 cells for one hour with or without methyl-β cyclodextrin (MβCD), a commonly used reagent to disrupt lipids rafts by depletion of cholesterol^33^, and then cultured them in the poly-HEMA-coated dishes to allow them to aggregate. Although cell aggregation was not changed by MβCD (Fig. 2A), the anoikis which is determined by Annexin V/PI staining was significantly increased in MβCD-treated cells at 24 hours (Fig. 2B). The similar results were obtained in the 4T1 mouse syngeneic TNBC cell line (Fig. 2C).These data suggest that lipid rafts disruption promotes aggregated cells to undergo anoikis. We have previously shown that cell aggregation activates EGFR^34^, which plays an important role in preventing cells from anoikis. Consistently, MβCD treatment reduced EGFR activation (indicated by the phosphorylation of EGFR at Y845, Fig. 2D&E) in both detached MDA-MB-231 and 4T1 cells. In addition, the active form of phosphorylated ERK was not changed, but phosphorylated p38 expression was increased in MβCD-treated cells (Fig. 2D&E), suggesting lipid rafts disruption also activates p38 mitogen-activated protein kinase activity to promote anoikis^35,36^. To further confirm that lipid rafts disruption can promote aggregated cells to undergo anoikis, we cultured MDA-MB-231 cells in the poly-HEMA-coated dishes in the absence or presence of simvastatin, which disrupts lipid rafts by *reducing the cholesterol content of lipid rafts*^37^. Similarly, simvastatin also promoted anoikis of aggregated cells (Fig. 2E). Taken together, these data suggest that disruption of lipid rafts induces aggregated cells to undergo anoikis.

### 2.3. Lipid raft integrity is required for Rac1 activation in aggregated cells to prevent anoikis.

**Figure 3:**
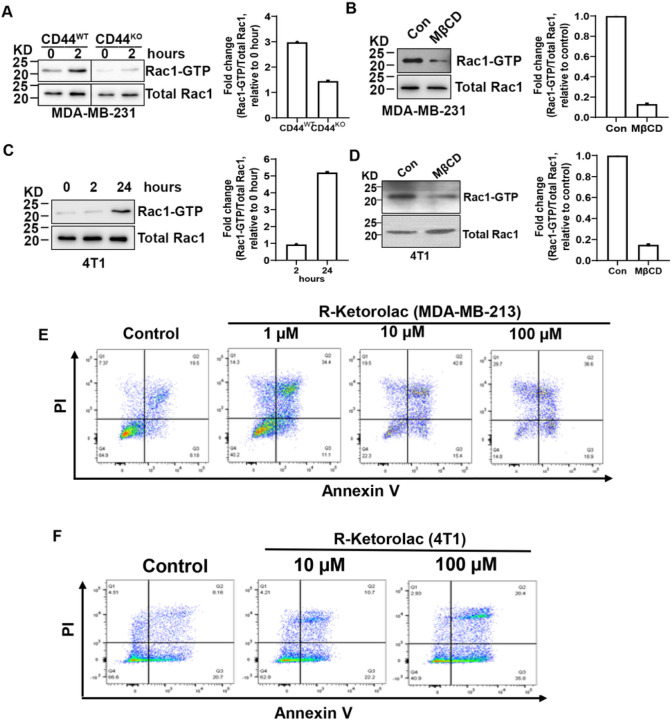
Lipid raft integrity is required for Rac1 activation in aggregated cells to prevent anoikis. **A.** Rac1 activation in CD44 ^WT^ MDA-MB-231 cells is dramatically increased comparing to CD44 ^KO^ MDA-MB-231 cells after 2 hours aggregation when cultured in poly-HEMA-coated dished (left). The levels of active Rac1-GTP are normalized with the levels of total Rac1 protein in cells, and the fold changes compared with 0 hour of each cell lines are calculated (right). The data represent one of two independent experiments. **B.** Lipid rafts disruption inhibits Rac1 activation in aggregated CD44^WT^ MDA-MB-231 cells. Cells were cultured in poly-HEMA-coated dished in the presence or absence of MβCD (5mM) for 2 hours, and then collected for Rac1 activity assay. The levels of active Rac1-GTP are normalized with the levels of total Rac1 protein in cells, and the fold changes compared with control are calculated (right). The data represent one of two independent experiments. **C.** Rac1 activation in 4T1 cells is dramatically increased when cultured in poly-HEMA-coated dished for 24 hours to aggregate (left). The levels of active Rac1-GTP are normalized with the levels of total Rac1 protein in cells, and the fold changes compared with 0 hour are shown (right). The data represent one of two independent experiments. **D.** Lipid rafts disruption inhibits Rac1 activation in aggregated 4T1 cells. The 4T1 cells were cultured in poly-HEMA-coated dishes to aggregate for 24 hours. To disrupt lipid rafts, aggregate cells were treated with MβCD (5mM) for the last 2 hours before collection. The levels of active Rac1-GTP are normalized with the levels of total Rac1 protein in cells, and the fold changes compared with control are calculated (right). The data represent one of two independent experiments. **E&F.** R-Ketorolac treatment promotes anoikis. The MDA-MB-231 cells (E) and 4T1 cells (F) were cultured in poly-HEMA-coated dishes in the presence or absence of R-Ketorolac for 48 hours, and the cells were collected for anoikis analysis by Annexin V/PI staining. The data represent one of two independent experiments.

Previously, we found that CD44-mediated cell aggregation activates Pak2^2,4^, which is a downstream target of Rac1^15^. Therefore, we investigated whether CD44-mediated cell aggregation activate Rac1. Indeed, Rac1 activity was dramatically increased in CD44 wild type (CD44^WT^), but not CD44 knockout (CD44^KO^) MDA-MB-231 cells after suspension cultured in poly-HEMA-coated dished for 2 hours (Fig. 3A), suggesting CD44-mediated cell aggregation activates Rac1. However, disruption of lipid raft integrity via MβCD inhibited Rac1 activation in aggregated CD44^WT^ MDA-MB-231 cells (Fig. 3B). In line with these findings, Rac1 activity was also increased in aggregated 4T1 cells after cultured in poly-HEMA-coated dished for 24 hours, but MβCD inhibited Rac1 activation (Fig. 3C&D). These data suggest that lipid raft integrity is required for Rac1 activation in aggregated cells. Since Rac1 activation can prevent anoikis^16,17^, and lipid rafts integrity is required for maintaining Rac1 membrane targeting and effector activation in ECM-detached cells^18^, we next examined the effect of Rac1 inhibition on anoikis. To this end, the cells were cultured in poly-HEMA-coated dishes, and treated in the absence or presence of racemic ketorolac (R-ketorolac), which has been identified as a robust Rac1 inhibitor^38,39^. We found that R-ketorolac significantly increased the anoikis of aggregated cells (Fig. 3E&F). Collectively, these data indicate that lipid raft integrity maintained by cell aggregation is required for Rac1 activation, which contributes to anoikis resistance.

### 2.4. CD44-mediated cell aggregation promotes CD44 cleavage and generates CD44 ICD.

**Figure 4:**
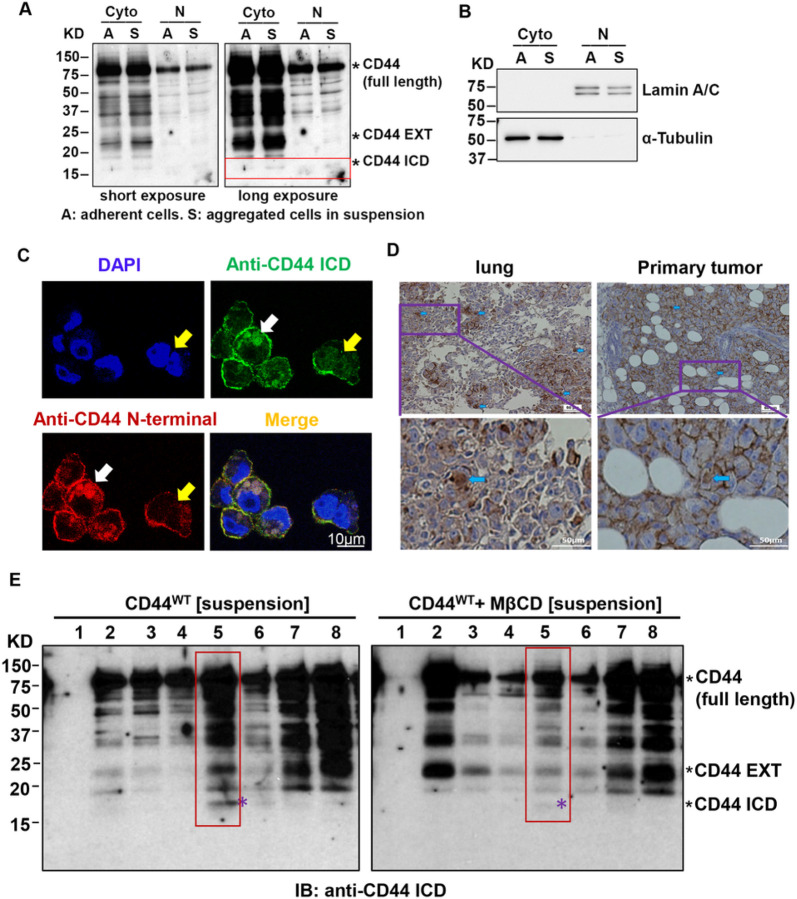
Cell aggregation promotes CD44 cleavage to generate CD44 ICD. **A.** CD44 ICD was increased in aggregated MDA-MB-231 cells after suspension culture in Poly-HEMA-coated dished for 24 hours. The cytoplasmic (Cyto) and nuclear (N) fractions were extracted for western blotting analysis using a specific anti-CD44 antibody which can detect CD44 ICD (17 kDa). The CD44 ICD was indicated in the red box. CD44 EXT refers to CD44 extracellular fragment. **B.** The purity of cytoplasmic and nuclei fractions was confirmed by α-tubulin and Lamin A/C expression, respectively. **C.** Representative IF staining shows CD44 ICD is localized in both cytosol (pointed with white arrow) and nuclei (pointed with yellow arrows). **D.** Nuclear CD44 ICD is present in both primary tumors and lung metastases of MDA-MB-231 tumor-bearing mice. Representative IHC staining shows nuclear CD44 ICD is present in both primary tumors and lung metastases (pointed with blue arrows) in MDA-MB-231 tumor-bearing mice. The enlarged images were shown in the lower panels. **E.**CD44 ICD is mainly generated in lipid rafts, but lipid raft disruption by MβCD reduces CD44 ICD generation. MDA-MB-231 cells were culture in Poly-HEMA-coated dished in the absence of presence of MβCD (10 mM) for 2 hours. The lipid rafts were isolated using Caveolae/Rafts Isolation Kit, and then used for western blotting analysis. The fracture 5 containing CD44 ICD was shown in red box, and indicated with purple star.

Proteolytic cleavages of CD44 is a key regulatory mechanism for the CD44-dependent signaling pathways^21,22^, leading to the release of CD44 ICD, which then translocate into the nucleus to regulate cancer stemness-related genes such as Oct4^26–28^. Since we have found that cell aggregation upregulates Oct4 expression^2^, we determined whether CD44 cleavage is induced by cell aggregation. Using a specific CD44 ICD antibody (Cosmo Bio), we found that CD44 ICD is increased in aggregated cells in both cytoplasm and nuclei (Fig. 4A&B). The IF staining further confirmed that CD44 ICD is generated in aggregated cells (Fig. 4C). In addition, the CD44 ICD was detected in both lung metastases and primary tumors of MDA-MB-231 tumor-bearing mice (Fig. 4D). Interestingly, CD44 ICD was mainly detected in lipid rafts-enriched faction 5 from aggregated cells. However, lipid raft disruption by *MβCD* dramatically reduces CD44 ICD generation in fraction 5 (Fig. 4E, red box). These data suggest that lipid raft integrity in aggregated cells promotes CD44 ICD generation.

### 2.5. CD44 and γ-secretase coexist at lipid rafts in aggregated cells, but are delocalized by lipid rafts disruption.

**Figure 5:**
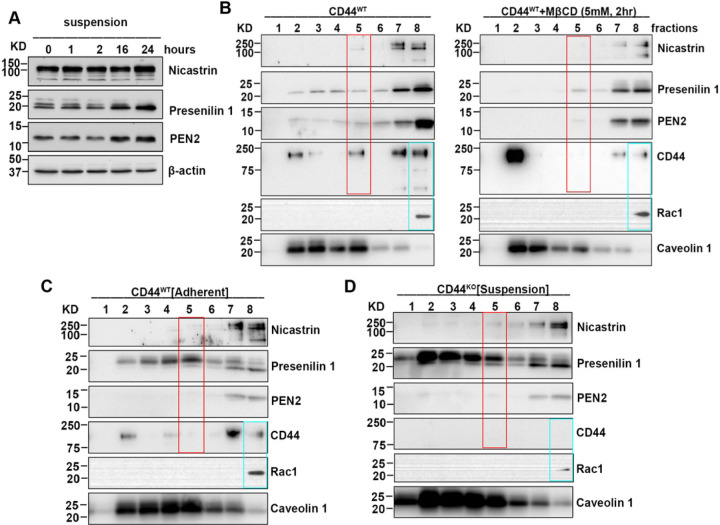
CD44 and γ-secretase coexist at lipid rafts in aggregated cells, but are delocalized by disruption of lipid rafts. **A.** The expressions of γ-secretase complex are increased during cell aggregation over time. The MDA-MB-231 cells were cultured in poly-HEMA-coated dishes for the indicated time points, and then collected for the western blotting analysis. **B.** CD44 and γ-secretase complex coexist at lipid rafts, but are delocalized by lipid rafts disruption. The MDA-MB-231 cells were cultured in poly-HEMA-coated dishes to aggregate for 24 hours. To disrupt lipid rafts, aggregate cells were treated with MβCD for the last 2 hours before collection. The lipid rafts were isolated using Caveolae/Rafts Isolation Kit, and then used for western blotting analysis. **C.** The expression of CD44, γ-secretase and Rac1 in adherent cultured CD44^wt^ MDA-MB-231 cells. The CD44^WT^ MDA-MB-231 cells were adherent cultured in cell culture dishes for 24. The lipid rafts were isolated using Caveolae/Rafts Isolation Kit, and then used for western blotting analysis. **D.** The expression of CD44, γ-secretase and Rac1 in suspension cultured CD44 ^KO^ MDA-MB-231 cells. The CD44^KO^ MDA-MB-231 cells were suspension cultured in poly-HEMA-coated dishes for 24 hours. The lipid rafts were isolated using Caveolae/Rafts Isolation Kit, and then used for western blotting analysis. The expression of CD44 and γ-secretase complex at fraction 5 was indicated in red box, and expression of CD44 and Rac1 at fraction 8 was shown in blue box. All data represent one of two independent experiments.

CD44 is cleaved by matrix metallopeptidases followed by γ-secretase to generate CD44 ICD^21–26^. Then we wondered whether γ-secretase could be involved in CD44 ICD generation during cell aggregation. Since γ-secretase is a multiprotein complex comprised of Presenilin, Nicastrin, Aph-1, and PEN2, all of which are essential for complete proteolytic activity, whereas the absence of even one results in the absence of γ-secretase activity^40–42^, we measured the expression of these proteins using γ-Secretase Antibody Sampler Kit (Cell Signaling Technology). Indeed, Presenilin, Nicastrin, and PEN2 were increased in aggregated cells (Fig. 5A). It is known that γ-secretase activity is predominantly localized in lipid rafts^31,43,44^. To further determine how γ-secretase is involved in CD44 ICD generation, we isolated lipid rafts from suspension-cultured MDA-MB-231 cells treated with or without *MβCD*, and examined the expression of γ-secretase complex by Western blot. Strikingly, CD44 and γ-secretase complex were co-expressed in lipid rafts-enriched fraction 5 in aggregated cells, but lipid rafts disruption by *MβCD* completely delocalized their co-expression in fraction 5, accompanied with CD44 moved to fraction 1, and Presenilin, Nicastrin, and PEN2 mainly detected in faction 7 and 8 (Fig 5B, red box). It is worth noting that the co-expression of CD44 and γ-secretase complex in fraction 5 was not observed in adherent-cultured MDA-MB-231 cells or suspension-cultured CD44^KO^ MDA-MB-231 cells (Fig. 5C&D), suggesting CD44-mediated cell aggregation induces CD44 and γ-secretase coexist in lipid rafts. Considering that CD44 ICD is mainly generated in fraction 5, which is inhibited by lipid raft disruption (Fig. 4D), these data suggest that lipid raft integrity maintains CD44 and γ-secretase co-expression in lipid rafts to promote CD44 ICD generation.

### 2.6. Disruption of lipid rafts inhibits cell aggregation-mediated metastasis.

**Figure 6:**
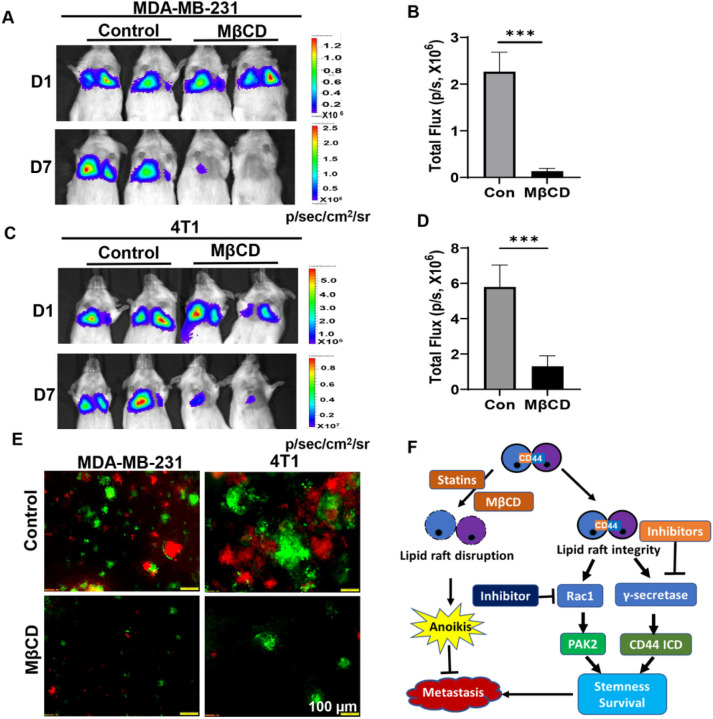
Disruption of lipid rafts inhibits cell aggregation-mediated metastasis. **A&B.** L2T-labelled CD44^WT^ MDA-MB-231 TNBC cells were cultured in poly-HEMA-coated dished for 24 hours, and then treated with or without 5mM MβCD for another 2 hours before injected into the NSG mice via tail vein. The representative BLI images of lung metastases were shown (A), and lung metastases at Day 7 (D7) were quantitated (B). Graph data were presented as mean ± SEM with n = 4 mice/group. T-test, *** p<0. 005. **C&D.** L2T-labelled CD44^WT^ 4T1 TNBC cells were cultured in poly-HEMA-coated dished for 24 hours, and then treated with or without 5mM MβCD for another 2 hours before injected into Balb/c mice via tail vein. The representative BLI images of lung metastases were shown (C), and lung metastases at Day 7 (D7) were quantitated (D). Graph data were presented as mean ± SEM with n = 4 mice/group. T-test, *** p<0. 005. **E.** Representative immunofluorescence images of lung metastasis of mice from A and C. **F.** Diagram of CD44-mediated cell aggregation maintains lipid raft integrity to prevent anoikis and enhance stemness to promote metastasis.

Cell aggregation via CD44 homophilic interaction after cell detachment enhances the metastatic ability of aggregated cell^2,4^. To further understand how cell aggregation promotes TNBC metastasis, we tested the effect of lipid rafts disruption on the metastatic ability of aggregated cells. MDA-MB-231 cells were cultured in poly-HEMA-coated dished for 24 hours to aggregate, and then treated with or without MβCD for another 2 hours before being injected into NSG mice via tail vein. The lung metastasis was significantly inhibited in MβCD-treated mice, compared to control mice (Fig. 6A, 6B & 6E). The similar results were also observed in 4T1 model (Fig. 6C–E). These data indicate that the lipid raft integrity of aggregated cells is important for their enhanced metastatic ability.

## 3. Discussion

Recently, we have found that cell aggregation via CD44 homophilic interaction enhances stemness, survival and metastatic ability of TNBC cells^2,34^. Here we further investigated the mechanism underlying enhanced metastatic ability of aggregated cells, and found that CD44-mediated cell aggregation maintains lipid raft integrity after cell detachment to prevent anoikis via Rac1 activation and enhance stemness by promoting CD44 cleavage. These data provide a new insight into the TNBC metastasis, and a potential therapeutic strategy to specially prevent CTC cluster-initiated TNBC metastasis by disrupting lipid raft integrity and blocking its-mediated downstream pathways such as Rac1 activation and CD44 cleavage.

Lipid rafts serve as a hub for the initiation of cellular signaling pathways by organizing the pathway components in ordered microdomains on the cell surface^11–13^. Cell detachment induces internalization of some lipid raft-related components, such as caveolin and cholesterol, and changes the activities of a variety of signaling molecules^45^. Consistently, our data showed that caveolin-1 was internalized into the cytoplasm in single cells after cell detachment. However, it was significantly reduced in aggregated cells. Anoikis is activated upon cell detachment. We previously found that CD44-mediated cell aggregation can prevent cells from anoikis after cell detachment^2^. Here, we further demonstrated that lipid rafts disruption either by *MβCD* or simvastatin promotes anoikis of aggregated cells. These data strongly suggest that lipid rafts integrity in aggregated cells is important for their survival. It is worth noting that simvastatin belongs to a group of drugs “statins” to lower cholesterol levels^46^, and several retrospective clinical studies have shown that statins reduce cancer mortality, recurrence and metastasis^47–52^. Our findings reveal a new mechanism about how statins can inhibit cancer metastasis, and suggest that statins could be more efficient eliminating CTC cluster-initiated metastasis. Future clinical trials are warranted to compare the response to statins between cancer patients with and without CD44^+^ CTC clusters.

It has been reported that cell aggregation inhibits anoikis after ECM-detachment in Her2 (ErbB2)-positive breast cancers^53^. In that study, it was shown that cell aggregation induces EGFR stabilization, and consequently activates the ERK survival pathway in an E-cadherin dependent manner in Her2-positive breast cancer cells^53^. In contrast, the MDA-MB-231 TNBC cell line used in our study does not express E-cadherin^2,54^, suggesting there is an E-cadherin independent cell aggregation mechanism in TNBC. Indeed, we have demonstrated that CD44 homophilic interaction-mediated cell aggregation is independent of its known ligand hyaluronan, E-cadherin, and other adhesion molecules^2,4^. Interestingly, we also found that CD44 stabilizes EGFR in TNBC cells^2^. Furthermore, CD44-mediated cell aggregation can directly activate EGFR^34^. It is worth noting that CD44-mediated cell aggregation does not activate ERK in the TNBC cell lines. Although disruption of lipid rafts promotes aggregated cells to undergo anoikis, it does not change the ERK activation (Fig. 2D & supplemental Fig. 1B). These data suggest that anoikis resistance in aggregated CD44^+^ TNBC cells are independent of ERK survival pathway. Instead, we found that disruption of lipid rafts activates p38, whose activation has been found can promotes anoikis^35,36^. These finding suggest that p38 inhibition could allow CTC to survive, and consequently promote metastasis. Indeed, studies have shown that p38 activation in breast cancer cells inhibits metastasis^55^. Since it is well accepted that p38 promotes tumor, the p38 inhibitors are undergoing clinical trials to treat cancer patients. Nevertheless, it should be cautious that these inhibitors may increase the chance of patients developing metastatic disease by inhibiting CTC anoikis.

γ-secretase inhibitors (GSIs) have been developed to inhibit γ-secretase cleavage in humans since γ-secretase was identified as a therapeutic target in Alzheimer’s disease (AD). However, several side effects were observed during clinical trials which led to discontinuation of the development of GSIs as therapeutic strategy against AD. One reason causing these side effects is that γ-secretase also cleave Notch^41^. Dysregulated Notch signaling has been directly linked to multiple cancers. Particularly, abnormal activation of Notch signaling plays a pivotal role in cancer stem cells maintenance and expansion^56,57^. Therefore, GSIs are being actively repurposed as anti-cancer therapeutics. In this study, we demonstrated that cell aggregation activates the γ-secretase complex and promotes CD44 cleavage to generate CD44 ICD. Since CD44 ICD can translocate to nuclear to activate stemness factors such as Oct4 to enhance tumor cell stemness and promote tumorigenesis^26–28^, our data provide a new insight into the mechanism of enhanced stemness properties and metastasis of aggregated cells. The findings also provide new preclinical evidence for repurposing GSIs in cancer patients, particularly patients with CD44^+^ CTC clusters. Whether cell aggregation could activate the Notch pathway also warrants further investigation.

CTCs have become one of the major focuses of translational cancer research, due to their easy accessibility for “liquid biopsy” with dynamic and live information on disease status. The presence of CD44^+^ CTC clusters correlates with a poor prognosis in breast cancer patients. Deeper understanding of the mechanism and signaling pathways mediating cell aggregation will lead to the development of new strategies to target CTC clusters and block cancer metastasis.

## 4. Materials And Methods

### 4.1. TNBC mouse models and animal studies.

Eight to ten-week-old female NSG (NOD SCID gamma), and Balb/c mice were purchased from Jackson Lab. All mice were housed in specific pathogen-free facilities, with normal chow diets and 12:12 hour light–dark cycle at 22°C in DLAR (Division of Laboratory Animal Resources) at the University of Kentucky. All animal procedures conformed to the National Institutes of Health Guide for the Care and Use of Laboratory Animals and were accepted by the University of Kentucky Institutional Animal Care and Use Committee. The human MDA-MB-231 TNBC cell line and mouse 4T1 TNBC cell line were transplanted into NSG mice and Balb/c mice, respectively. Both MDA-MB-231 and 4T1 breast cancer cells were labeled with L2T as described previously^2^.

To analyze lung metastasis using Bioluminescence imaging (BLI), the mice were injected intraperitoneally with 100 μL of D-luciferin (30 mg/mL, Gold Biotechnology). After 5 min, mice were anesthetized with isoflurane, and bioluminescence images were acquired using the Xenogen IVIS Spectrum system (Caliper Life Sciences, Waltham, MA). Acquisition times ranged from 5 s to 5 min. Signals are presented as total photon flux and analyzed using Living Image 3.0 software (Caliper Life Sciences). At the end point, the lungs were also removed and imaged by fluorescence microscopy.

### 4.2. Cell culture and treatment

MDA-MB-231 and 4T1 cells were obtained from ATCC. Early passage of cells (<20 passages) was maintained in DMEM with 10% FBS + 1% penicillin-streptomycin (P/S) at 37 °C in an atmosphere of 5% CO_2_. CD44 KO MDA-MB-231 cells were generated as described previously^2^. For suspension cell culture, cells were trypsinized into single cells and then seeded to poly-hydroxyethyl methacrylate (poly-HEMA, Sigma-Aldrich, St. Louis, MO)-coated plates and treated with or without Methyl-β-cyclodextrin (M-βCD, Sigma-Aldrich, C4555), simvastatin (Sigma-Aldrich, S6196) and R-Ketorolac (MedChemExpress, Monmouth Junction, NJ, HY-B0580B).

### 4.3. Caveolae/rafts isolation

Lipid rafts fractions were isolated by a commercial caveolae/rafts isolation Kit (Sigma-Aldrich, CS0750) according to the instruction. Briefly, cells were washed twice with ice-cold PBS and lysed using lysis buffer containing 1% Triton X-100 and 1% protease inhibitor cocktail for 30 min on ice. The density gradients were prepared at 35%, 30%, 25%, 20% and 0% concentrations using the recommended amounts of the cell lysate, lysis buffer and OptiPrep medium, and then centrifuged at 200,000g for 4 h at 4 °C using a TH-641 rotor (WX+ ultracentrifuge, Thermo Scientific, Waltham, MA). Each fraction was carefully collected from top to bottom of the ultracentrifuge tube, and transferred to a microcentrifuge tube. The fractions were analyzed by western blot.

### 4.4. Extraction of nuclear and cytoplasmic fractions

Cells were suspension cultured in Poly-HEMA-coated dished for 24h, and then the nuclear and cytoplasmic fractions were extracted using a commercial nuclear extraction kit (Active Motif, Carlsbad, CA, 40010) according to the manufacturer’s instructions, followed by Western blotting analysis. The purity of cytoplasmic and nuclei fractions was confirmed by α-tubulin and Lamin A/C expression, respectively.

### 4.5. Western blotting

Cells were lysed by RIPA buffer with protease inhibitor cocktail for 30 min on ice, then centrifuged for 12 min at 4 °C. Protein concentration was measured through BCA Protein Assay Kit (Thermo Fisher, 23227), and equal amounts of protein of each sample (10–40 μg) were separated by SDS-PAGE and then transferred to nitrocellulose membranes. After blocking with 5% non-fat dry milk in 0.1% tween/TBS (Tris-buffered saline) for 1 h, the membrane was washed and incubated with primary antibody overnight at 4 °C. The appropriate HRP-conjugated second antibody was added after washing with 0.1% tween/TBS. The transferred protein was detected using the clarity western ECL reagent (Bio-Rad, Hercules, CA, 1705061), a Bio-Rad ChemiDoc imaging system or X-ray film. Primary antibodies used were Rac1 (1:500, Cytoskeleton, Denver, CO, ARC03), CD44 (1:2000, Thermo Fisher, MA5–15462), CD44 ICD (1:1000, COSMO BIO USA, Carlsbad, CA, KAL-KO601), p-EGFR (Tyr845) (1:1000, Cell Signaling, Danvers, MA, 2231), EGFR (1:1000, Santa Cruz, Dallas, TX, sc-03), p-p38 (Thr180/Tyr182) (1:1000, Cell Signaling, 4511), p38 (1:1000, Cell Signaling, 9212), p-ERK1/2 (Thr202/Tyr204) (1:1000, Cell Signaling, 4370), ERK1/2 (1:1000, Santa Cruz, sc-514302), Caveolin 1 (1:5000, Sigma-Aldrich, C3237), Lamin A/C (1:1000, Cell Signaling, 4777), α-Tubulin (1:4000, Sigma-Aldrich, T5168), Nicastrin (1:1000, Cell Signaling, 5665), Presenilin1 (1:1000, Cell Signaling, 5643), PEN2 (1:1000, Cell Signaling, 8598), β-Actin (1:1000, Thermo Fisher, MA5–11869). Secondary antibodies used were goat polyclonal anti-mouse (IgG) HRP (1:10000) (Thermo Fisher), and goat polyclonal anti-rabbit (IgG) HRP (1:10000) Thermo Fisher).

### 4.6. Rac1 pulldown activation assay

Rac1 activity was carried out using a Rac1 Pull-Down Activation Assay Biochem Kit (Cytoskeleton, BK035) according to the instruction. Briefly, cells were lysed, and equivalent amounts of proteins lysates were incubated with PAK-PBD beads at 4°C for 1 h. Beads were washed with lysis buffer, and precipitated Rac1-GTP was analyzed by SDS-PAGE and Western blotting. Rac1-GTP levels were normalized to total Rac1 by densitometric analysis with ImageJ software.

### 4.7. Immunofluorescence

Cells were cultured in poly-HEMA-coated dishes to aggregate for 24 hours. Afterward, cells were collected and spun onto Fisherbrand^™^ Superfrost^™^ Plus microscope slides (Thermo Fisher), and fixed with 4% paraformaldehyde for 10 min. Cells were permeabilized using 0.25% Triton X-100 in PBS, followed by blocking with 2% BSA in PBS for 1 hour. Caveolin 1, CD44 (N-terminal), and CD44-ICD primary antibodies were then incubated with cells overnight at 4 °C. Cells were then washed with PBS and incubated with Alexa Fluor^™^ 488 and Alexa Fluor^™^ 568-conjugated secondary antibodies (Thermo Fisher) for 1 hour, and finally, nuclei were counterstained with DAPI. The images were taken on a confocal microscope (Nikon Confocal, Melville, NY).

### 4.8. Immunohistochemistry

Primary tumors and lung tissues from tumor-bearing mice were paraffin-embedded and sectioned by routine techniques. Deparaffinization and rehydration of tissue sections were first achieved. Heat-induced antigen retrieval was done using Antigen Unmasking Solution (Vector Laboratories, Newark, CA, H-3300–250) for 15–20 min. Tissue sections were blocked with TBS/5% BSA, and incubated with CD44-ICD primary antibody overnight at 4 °C, followed by DAB staining. Sections were counterstained with hematoxylin.

### 4.9. Anoikis analysis

Poly-HEMA was reconstituted in 95% ethanol to a concentration of 20 mg/mL. To prepare poly-HEMA-coated plates, poly-HEMA solution was added to cell culture plates/dishes and allowed to dry overnight in a tissue culture hood. Cells were cultured in poly-HEMA-coated plates/dishes as indicated time points, and the anoikis was analyzed by annexin V/PI staining (Pacific Blue^™^ Annexin V Apoptosis Detection Kit with PI, BioLegend, San Diego, CA) according to the manufacturer’s instructions.

### 4.10. Statistical analysis

Student’s t-test was performed for the statistical analyses between 2 samples as appropriate using GraphPad Prism software. One-way ANOVA (followed by Tukey post-hoc test) was performed to analyze differences among multiple groups. Data are presented as mean ± SD from at least three biological replicates, and p<0.05 was considered significant.

## Data Availability

The data that support the findings of this study are available from the corresponding author upon reasonable request.
